# Comprehensive Characterization of Immune Landscape Based on Epithelial-Mesenchymal Transition Signature in OSCC: Implication for Prognosis and Immunotherapy

**DOI:** 10.3389/fonc.2021.587862

**Published:** 2021-07-01

**Authors:** Si-yuan Zhang, Xian-yue Ren, Chun-yang Wang, Xi-juan Chen, Ruo-yan Cao, Qin Liu, Xue Pan, Jia-ying Zhou, Wei-lin Zhang, Xin-Ran Tang, Bin Cheng, Tong Wu

**Affiliations:** ^1^ Guangdong Provincial Key Laboratory of Stomatology, Guanghua School of Stomatology, Hospital of Stomatology, Sun Yat-sen University, Guangzhou, China; ^2^ Department of Radiation Oncology, Nanfang Hospital, Southern Medical University, Guangzhou, China

**Keywords:** oral squamous cell carcinoma, epithelial to mesenchymal transition, tumor microenvironment, prognosis, immune checkpoint

## Abstract

Current anatomic TNM stage classification fails to capture the immune heterogeneity of oral squamous cell carcinoma (OSCC). Increasing evidence indicates the strong association between epithelial-mesenchymal transition (EMT) and tumor immune response. In this study, we employed an EMT signature to classify OSCC patients into epithelial- (E-) and mesenchymal- (M-) phenotypes using TCGA and GSE41613 transcriptome data. The ESTIMATE and CIRBERSORT analyses implied that the EMT signature genes originated from the stroma of the bulk tissue. The M-subtype tumors were characterized as “immune-hot” with more immune cell infiltration than the E-subtype ones. The low infiltration of active immune cells, the high infiltration of inactive immune cells, and the high expressions of immune checkpoints demonstrated an immunosuppressive characteristic of the M-subtype tumors. Moreover, we developed and validated a novel prognostic classifier based on the EMT score, the expressions of seven immune checkpoints, and the TNM stages, which could improve the prediction efficiency of the current clinical parameter. Together, our findings provide a better understanding of the tumor immune heterogeneity and may aid guiding immunotherapy in OSCC.

## Introduction

Oral squamous cell carcinoma (OSCC), arising from the mucosal lining of the buccal mucosa, floor of the mouth, tongue, and other parts within the oral cavity, is a heterogeneous subgroup of head and neck squamous cell carcinoma (HNSC) (1, 2). Over the past decades, patients with advanced OSCC received platinum-based chemotherapy and best supportive care. Prognostic definition and treatment decisions of OSCC patients are mainly dependent on the tumor-node-metastasis (TNM) classification. However, only 50% of patients are expected to survive over five years and continue to have a very poor prognosis (2, 3). Hence, the current anatomical-based staging system is not sufficient to select patients at high risk of treatment failure. Identifying the novel biomarkers which can reflect tumor heterogeneity is still a challenge.

Despite the fact that the immune system should reject cancer cells as ‘foreign’ automatically, cancer cells are usually recognized as ‘self’. The natural balance between cancer and the immune system is tolerance, which could be sustained by diverse mechanisms, including the reduced and dysfunctional regulatory immune cells (especially T cells), the abnormally expressed chemokines and cytokines, and dysregulated immune checkpoint pathways (4, 5). Given the encouraging results obtained from the anti-PD-1 antibodies therapy in many cancers, recurrent and metastatic HNSCC (R/M-HNSCC) patients with refractory treatment were allowed to use anti-PD1 therapies (pembrolizumab or nivolumab) by the America FDA (6–9). Considerably, only a minority of patients responded to the anti-PD1 therapies. Understanding the intrinsic resistance mechanisms and identifying the novel therapeutic targets of immune-based therapies have been put on the urgent agenda (10, 11).

Epithelial-mesenchymal transition (EMT), a phenotypic shift along the epithelial-mesenchymal axis, is orchestrated by a spectrum of transcription factors, like SNAILs, ZEBs, and TWISTs. EMT is enrolled in cancer stem cell maintenance, tumor metastasis, and therapeutic resistance, and has been broadly established in cancer initiation and progression, including OSCC (12–15). Increasingly, the relationships between EMT and immune response have been noticed (16). EMT was reported to upregulate the expression of immune checkpoints, like PD-L1, which altered the balance of infiltrating immune cells and induced immune suppression (17–19). Inversely, PD-L1 could promote EMT in several cancers, such as esophageal cancer, nasopharyngeal carcinoma, and glioblastoma (20, 21). Furthermore, the expressions of EMT markers were identified to be closely associated with immune checkpoints expression and cancer patients’ survival (13, 22). However, in metastatic melanoma, mesenchymal and inflammatory tumor phenotypes might be associated with innate anti-PD-1 resistance (23). Thus, the association of EMT and immune activity in cancer is controversial and needs to be further elucidated.

In OSCC, Hirai, M et al. found that *in vitro* co-culturing with mesenchymal tumor cells upregulated PD-L1 expression on tumor-associated macrophages and dendritic cells. However, the relationship between EMT and the OSCC immune landscape is still unknown. Furthermore, there are several questions that need to be addressed: Is there an EMT signature which could be used to classify OSCC tumors? What is the cellular origin of EMT signature genes in OSCC bulk tumors? What are the differences of immune landscapes between EMT subtypes in OSCC? Is there a prognostic model based on EMT signature and immune components which could improve the prediction efficiency of the TNM staging system in OSCC?

Here, we developed and confirmed a three-subtype classification according to the EMT signature genes using the OSCC cohorts from TCGA and GSE41613 datasets. The biological roles and immune landscapes in EMT subtypes were addressed. A combined pattern of EMT score, immune checkpoints, and TNM stage was identified as a reliable prognostic classifier for OSCC patients. Therefore, a better understanding of the EMT subtypes may aid in guiding the ongoing clinical research on OSCC immuno-oncology.

## Methods

### Clinical Material and Patient Sample Characteristics

A total of 413 patients from The Cancer Genome Atlas (TCGA, n = 315) and GSE41613 (n = 97) (24) were included in this study. Samples with at least 50% expressed genes were enrolled for analysis. Gene expression data and clinical data were downloaded from the TCGA data portal (https://tcga-data.nci.nih.gov/tcga/, accessed September 19, 2019) and Gene Expression Omnibus data portal (https://www.ncbi.nlm.nih.gov/geo/). Formalin-fixed paraffin-embedded (FFPE) OSCC tissues were obtained from patients treated at the Hospital of Stomatology, Sun Yat-sen University (Guangzhou, China). Informed consent was obtained from all patients and the study was approved by the Medical Ethics Committee of the Hospital of Stomatology, Sun Yat-sen University.

### Generation of EMT Score

For each sample, an EMT score was calculated by an averaging scheme based on the mRNA expression of 77 genes previously published by Milena P. Mak et al. (25). The scores were computed as the average expression level of “mesenchymal” genes minus “epithelial” genes. Samples were then classified by EMT score as epithelial (E-) subtype (EMT scores ≤ lowest 1/3) or mesenchymal (M-) subtype (defined by EMT scores ≥ highest 1/3) in both TCGA and GSE41613 datasets.

### Gene Enrichment Analyses

The gene set enrichment analysis (GSEA) and GO enrichment analyses were performed using the R package cluster profiler (26). The hallmark gene sets (including 50 gene sets) and KEGG subset of the Canonical Pathways collection (including 186 gene sets) of GSEA were downloaded from the GSEA website (https://www.gsea-msigdb.org/gsea/msigdb/collections.jsp) and performed. Gene sets with a false discovery rate (FDR) < 0.05 after performing 1000 permutations were considered to be significantly enriched. The categories (molecular function, biological processes, and cellular component) of GO terms were performed; an FDR < 0.05 was considered to be statistically significant. The ssGSEA was performed using the R package GSVA with the reported gene sets (**Supplementary Table 2**) (27). The enrichment score across samples were normalized using Z-score.

### ESTIMATE Analysis

The ESTIMATE was an algorithm designed by Yoshihara et al. to predict tumor purity using gene expression data (28). For the TCGA OSCC dataset, the scores (immune score, stromal score, and ESTIMATE score) were downloaded from the ESTIMATE portal website (http://bioinformatics.mdanderson.org/estimate/). For the GSE41613 dataset, the scores were computed by R package ESTIMATE using gene expression data based on the Affymetrix platform.

### Immunohistochemistry and Image Analysis

The tissue sections from OSCC patients were deparaffinized, rehydrated, and the endogenous peroxidase activity was blocked with 0.3% hydrogen peroxide for 15 min. Then, 10% BSA was used for 10 min to block nonspecific binding. Subsequently, the tissue sections were incubated with anti-human CD8a antibodies (1:5000, Proteintch) or anti-human CDH1 (1:1500, Proteintech) anti-bodies at 4°C overnight and then incubated with biotinylated secondary antibody for 30 min at room temperature. Thereafter, the tissue sections were reacted with streptavidin-peroxidase conjugate and 3,3’-diaminobenzidine to detect and visualize the staining. All the tissues were digitally scanned at 200x magnification into high-resolution digital images using a pathology scanner (Aperio AT Turbo, Leica Biosystems). The images were visualized using the Aperio ImageScope software program and analyzed with the Aperio Image Toolbox and GENIE analysis tool. The densities of immune cells expressing CD8a were evaluated using the Aperio cytoplasmic algorithm, and counting the cells positive for them in five square areas (1 mm^2^ each) in intertumoral compartments. The H-score was calculated by the staining intensity (0: no staining; 1: weak, light yellow; 2: moderate, yellow-brown; 3: strong, brown) and the proportion of positive cells (score 2: 1, <10%; 2, 10%–35%; 3, 35%–70%; 4, >70%).

### CIBERSORT Analysis

The CIBERSORT method with the LM22 gene signature from CIBERSORT website (https://cibersort.stanford.edu/) (29) was used to estimate the relative proportions of 22 immune cell subsets in order. The results were filtered by P < 0.05.

### Construction of EIT Prognostic Model

The multivariate Cox regression analysis through the backward stepwise approach based on the mRNA levels of 19 immune checkpoint genes was employed to select the independent prognostic factors of overall survival. Then, the EMT score, seven immune checkpoints, and TNM stage were combined to develop the EIT model, the risk score was weighted by their regression coefficient and computed for each patient using the formulas as follows: Risk score (TCGA dataset) = (0.340169595×expression of PD-L1) -(0.193147995×expression of PD-L2)-(0.279020671×expression of CTLA4) - (0.204675492×expression of CD28) + (0.324729962×expression of TIM3) + (0.029264054×expression of OX40L) + (0.140068210×expression of VISTA) - (0.009029925 × EMT score) + (0.745233185×TNM stage); Risk score (GSE41613 dataset) = (0.71446977×expression of PD-L1) + (0.07393616×expression of PD-L2)-(0.75801486×expression of CTLA4) + (0.52419494×expression of CD28) - (0.09773648×expression of TIM3) - (0.67053261×expression of OX40L) + (0.39243416×expression of VISTA) - (0.66827169 × EMT score) + (1.66367832×TNM stage). The cut-off values were set by maximum AUC of the log-rank test for overall survival.

### Statistical Analysis

Statistical analyses were done in R (version 3.6.1) and SPSS (version 20.0). Overall survival was calculated using the Kaplan-Meier method with the log-rank test and univariate and multivariate Cox regression analyses. The Fisher exact tests were used for categorical variables. The cut-off value was set by maximum AUC. Significance was defined as *P* < 0.05.

## Results

### Constructing an EMT-Subtype Classifier Based on the EMT Signatures in OSCC

**Figure 1:**
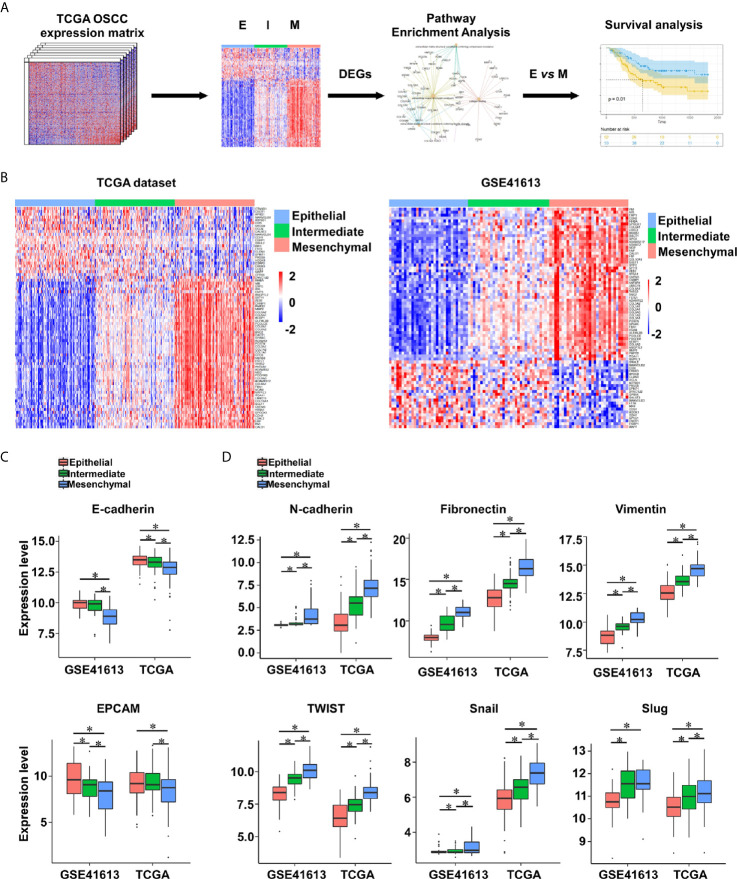
The EMT subtypes are constructed in OSCC. **(A)** The analytic pipeline in this study. **(B)** Heatmap of EMT signature gene expression levels in OSCC tumors from the TCGA (n = 315) and GSE41613 (n = 97) datasets. **(C, D)** The expression levels of epithelial markers (E-cadherin and EPCAM) **(C)** and mesenchymal markers (N-cadherin, fibronectin, vimentin, SNAIL, SLUG, and TWIST) **(D)** in OSCC tumors. ^*^ *p* value < 0.05, mean ± s.d., student’s *t*-test.

To explore the impact of EMT on intrinsic heterogeneous in OSCC, the EMT signature (25) was applied to calculate the EMT score of each OSCC specimen from the TCGA cohort (n = 315) and GSE41613 dataset (n = 97). Genes of the EMT signature are shown in **Supplementary Table 1**. OSCC patients were evenly divided into three subtypes according to the EMT scores: epithelial-subtype (E-subtype), intermediate-subtype (I-subtype), and mesenchymal-subtype (M-subtype) (**Figures 1A, B**). The expression levels of epithelial markers (E-cadherin and EPCAM) were reduced from the E-subtype to M-subtype, while the mesenchymal markers (N-cadherin, fibronectin, vimentin, SNAIL, SLUG, and TWIST) had the reverse expression patterns (**Figures 1C, D**).

**Figure 2:**
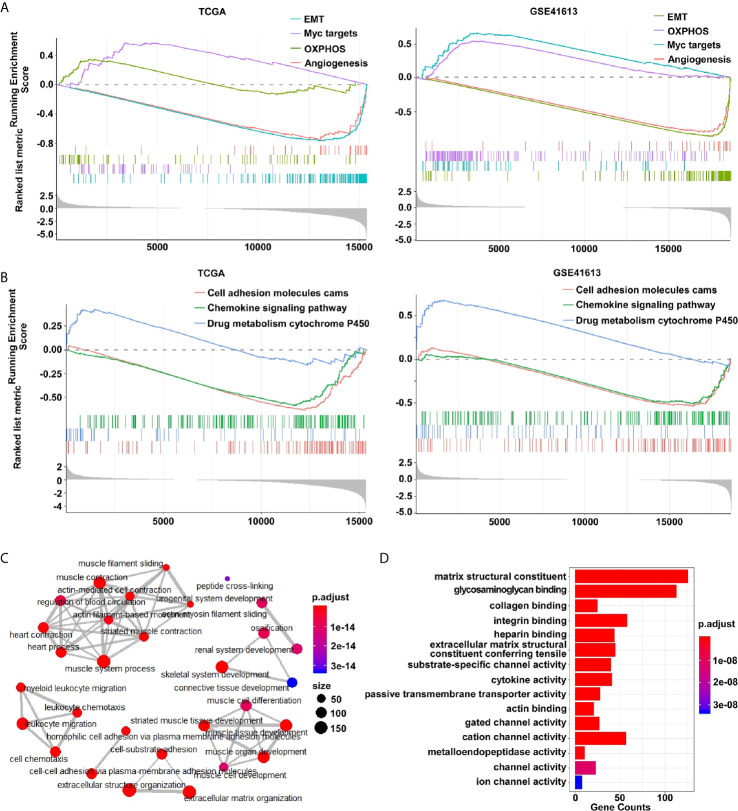
The differentially expressed genes between E- and M-subtypes are enriched in EMT- and immune-associated signaling pathways in OSCC. **(A)** The hallmark modular and **(B)** the KEGG modular of GSEA analysis, and **(C)** the biological process modular and **(D)** the molecular function modular of Gene Ontology analysis show the DEGs are enriched in EMT-associated and immune-associated signaling pathways.

The hallmark modular of gene set enrichment analysis (GSEA) was performed to investigate the biological functions of differentially expressed genes (DEGs) between E-subtype and M-subtype. As expected, the DEGs were enriched in the EMT, OXPHOS (oxidative phosphorylation), MYC targets, and angiogenesis in both TCGA and GSE41613 datasets (**Figure 2A**). The KEGG (Kyoto Encyclopedia of Genes and Genomes) modular of GSEA analysis indicated that the DEGs participated in cell adhesion molecules (CAMs) (**Figure 2B**). The cellular component of Gene Ontology (GO) analysis found that the collagen-containing extracellular matrix genes and extracellular matrix genes were differentially expressed between E- and M-subtypes (**Supplementary Figure 1**). Biological process indicated that the DEGs were significantly associated with collagen, integrin glycosaminoglycan binding, and extracellular structural constituent, while molecules function analysis implied that DEGs were enriched in matrix structural constitute, integrin binding, and extracellular matrix structure (**Figures 2C, D** and **Supplementary Figure 2**). Notably, the DEGs between E- and M-subtypes were substantially enriched in the chemokine signaling pathway, leukocyte chemotaxis and migration, and cytokine activity (**Figures 2B–D** and **Supplementary Figures 1**, **2**), indicating a diverse immune microenvironment in each subtype. Collectively, these findings confirmed the successful establishment of a three-subtype classification based on the EMT signatures in OSCC.

### The EMT Signature Is Negatively Linked to the Tumor Purity in OSCC

**Figure 3:**
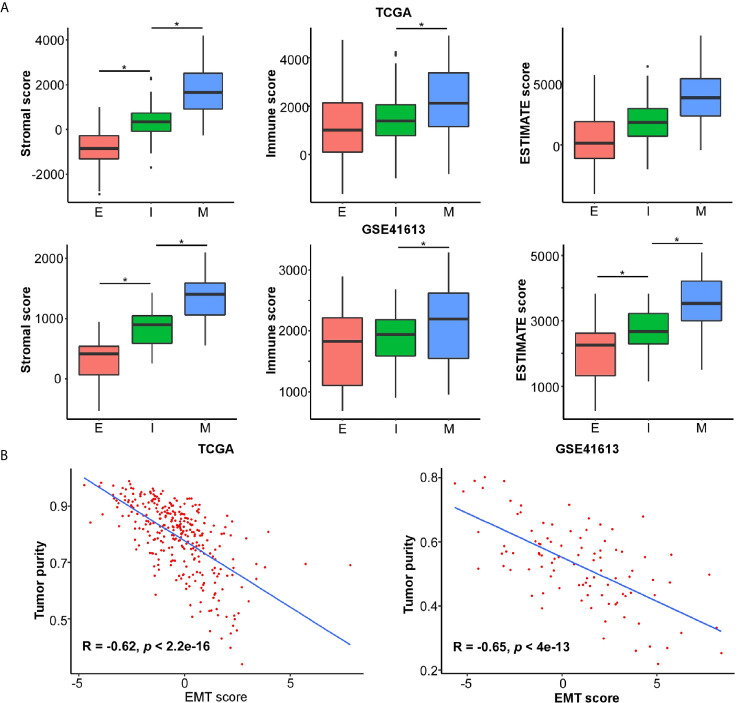
EMT signature is negatively associated with tumor purity and likely emanates from stroma in OSCC. The ESTIMATE method is applied to determine the cellular components and tumor purity. **(A)** The stromal score, immune score, and ESTIMATE score in each EMT subtype. ^*^ *p* value < 0.05, mean ± s.d., student’s *t*-test. **(B)** The correlations of EMT score and tumor purity in OSCC. The *R* and *p* values were calculated using Pearson correlation coefficient.

**Figure 4:**
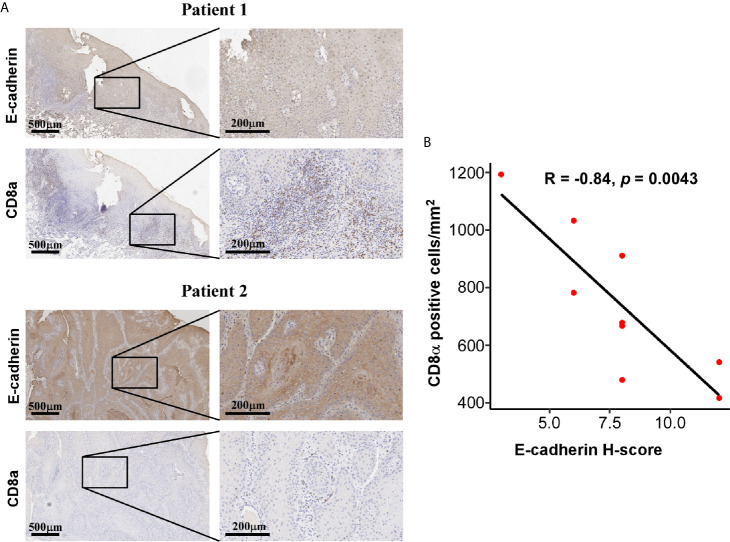
Expression of E-cadherin and CD8a was negatively correlated. **(A)** Representative IHC images showing a negatively correlated expression pattern of E-cadherin and CD8a in OSCC tissue. IHC images were photographed at 50 × and 200 × magnification. Brown represents positive E-cadherin or CD8a staining, respectively, while blue represents the nucleus. **(B)** The correlations of CD8a positive cells per mm^2^ and E-cadherin H-score in OSCC tissues. The *R* and *p* values were calculated using Spearman correlation coefficient.

The application of single cell RNA-seq (scRNA-seq) in HNSC demonstrated that the mesenchymal subtype was found exclusively in nonmalignant cells but not in the malignant cells, suggesting that the mesenchymal signature genes might be an indicator of the high content of the tumor microenvironment (TME) (30). To explore the compositional differences of cell components in the E- and M-subtypes, the ESTIMATE method, which was applied to estimate the proportion of stromal and immune components and tumor purity from the bulk transcriptomes, was employed (28). The results revealed that the stromal component and immune infiltration in the M-subtype tumors were higher than those in the E-subtype tumors (**Figure 3A**). The expression levels of the EMT signature genes were negatively correlated with tumor purity (**Figure 3B**). Then, we analyzed the correlation between the expression levels of E-cadherin and the spatial distribution pattern of CD8+ T cells in our OSCC specimen. We found that patients with higher E-cadherin levels exhibited a lower level of CD8+ T cell infiltration (r = -0.85, p = 0.004, **Figure 4**). Thus, these findings demonstrated that M-subtype tumors were inflamed, and the EMT signature genes might originate from stromal cells in the TME rather than epithelial cancer cells in OSCC.

### EMT Subtypes Are Associated With Distinct Immune Landscapes

**Figure 5:**
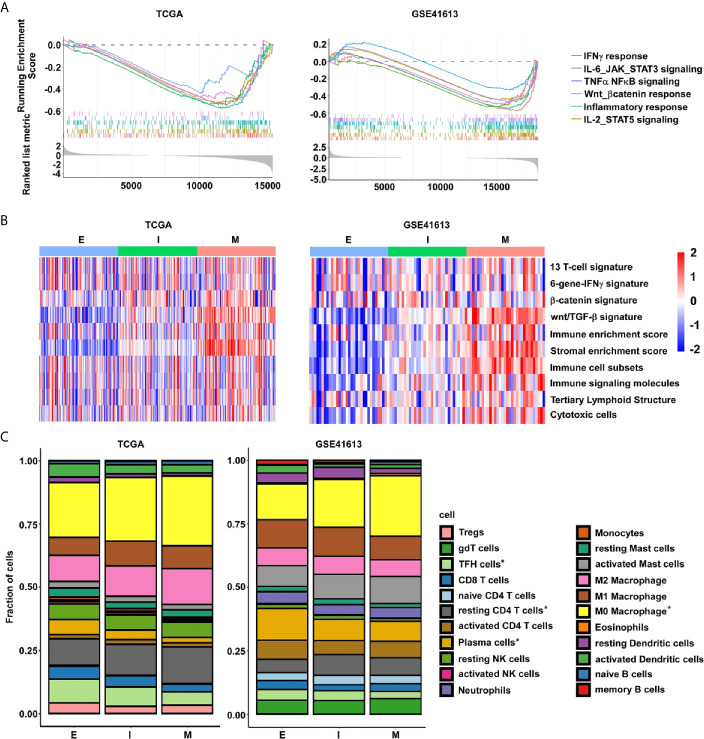
The M-subtype OSCC is highly inflamed and is associated with an immunosuppressive TME. **(A)** The enrichment of immune related signaling in M-subtype OSCC are determined by GSEA analysis. **(B)** The enrichment of immune-related gene signatures in each EMT subtype tumor is performed by ssGSEA analysis. Red, high ssGSEA scores; blue, low ssGSEA scores. **(C)** The lymphocytes fractions of each EMT subtype were calculated by CIBERSORT. ^*^ *p* value < 0.05, student’s *t*-test.

Given the increasing evidence that show the essential roles of EMT in developing antitumor immunity and response, we then focused our insight on to the differences in immune landscapes between the E- and M-subtypes. The GSEA analysis identified that immune-inflammatory response-associated signaling, including IFN-γ response signaling, IL6/JAK/STAT3 signaling, IL2/STAT5 signaling, TNF-α/NF-κb signaling genes, and inflammatory response signaling, were highly enriched in M-subtype tumors (**Figure 5A**). To further investigate the immune-related gene signatures that represents different immune status in EMT subtypes, single sample gene set enrichment analysis (ssGSEA) was performed (**Supplementary Table 2**). We confirmed that patients with mesenchymal class had increased stromal enrichment score and Wnt/β-catenin signature. Furthermore, the signatures identifying immune cytolytic activity, such as immune cell subsets, cytotoxic cells, and tertiary lymphoid structure (TLS) were substantially higher in M-subtype tumors than E-subtype ones. Inspiringly, the six-gene IFNγ signature which was previously reported to predict for pembrolizumab response in HNSC was also highly enriched in M-subtype patients (**Figure 5B**), implying that M-subtype patients might be more sensitive to the anti-PD1 therapy than E-subtype ones.

Next, to systematically map compositional differences of immune cell components in the E- and M-subtypes, the CIBERSORT method, which could infer the relative proportions of 22 distinct functional immune cells subsets and rule out the influence of tumor cells in the bulk samples based on the gene expression profiles, were employed (29). In both datasets, macrophages and T cells were the top two predominant immune cell types in either E- or M-subtype OSCC. However, the results indicated a striking difference in the proportions of leukocyte compositions in each subtype. In the TCGA database, the infiltration fractions of macrophages (unstimulated M0 and anti-inflammatory M2) and resting CD4 memory T cells were obviously upregulated, while active T cells (e.g., cytotoxic CD8, activated CD4 memory cells, and follicular helper cells), activated dendritic cells (DCs), and plasma cells (PCs) were obviously downregulated in M-subtype tumors than E-subtype ones. In the GSE41613 dataset, in contrast to the E-subtype tumors, the infiltration fractions of M0 macrophages and resting CD4 memory T cells were significantly higher, while the follicular helper T cells (TFH cells), resting DCs, memory B cells, and PCs were significantly lower in M-subtype tumors. Collectively, higher fractions of inactive immune cells (M0 macrophages and resting memory CD4+ T cells), and lower fractions of active immune cells (TFH cells and PCs) were seen in M-subtype tumors in both cohorts (**Figure 5C** and **Supplementary Table 2**). Therefore, these data illustrated that tumors in different EMT subtypes exhibited distinct immune responses. M-subtype OSCC might have a more suppressive tumor immune microenvironment than E-subtype OSCC.

### The Distinct Immune Checkpoints Expression Profiles in EMT Subtypes

**Figure 6:**
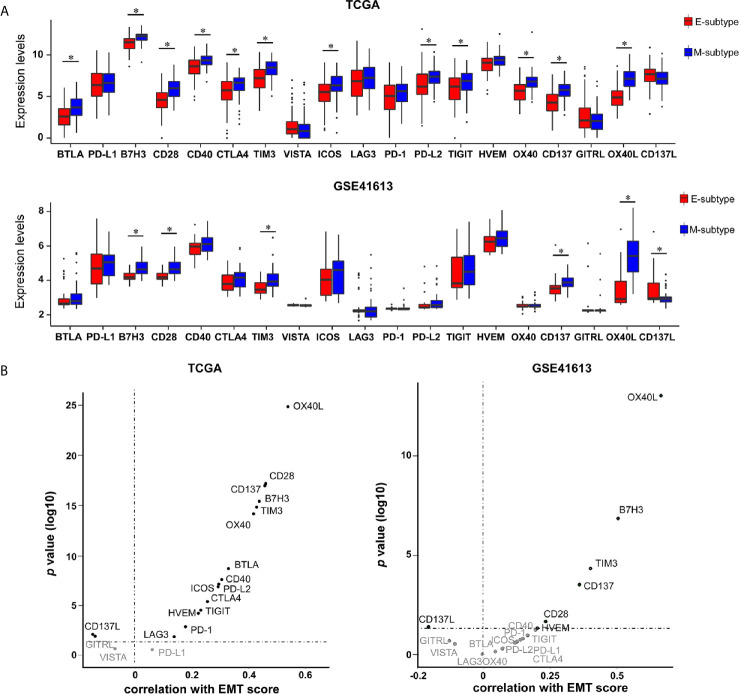
The expression levels of immune checkpoints are elevated in M-subtype OSCC. **(A)** The mRNA levels of 19 immune checkpoints in E- and M-subtypes. **(B)** The correlation of EMT scores and 19 immune checkpoints expression levels. ^*^ *p* value < 0.05, mean ± s.d., student’s *t*-test.

Immune checkpoints are well known in helping tumor cells escape from immune surveillance in various cancers, including OSCC. So far, several immune checkpoint targets have been transferred from the laboratory to clinical application, including 11 co-inhibitors (B7H3, BTLA, CTLA4, HVEM, LAG3, PD-1, PD-L1, PD-L2, TIM-3, TIGIT, and VISTA) and 8 co-stimulators (CD28, CD40, CD137, CD137L, GITRL, ICOS, OX40, and OX40L) (25, 31, 32). To identify potential therapeutic biomarkers in each EMT subgroup, the expression profiles of those immune checkpoints were assessed. We noticed a substantial upregulation of multiple immune checkpoints in M-subtype tumors in contrast to those in E-subtype tumors. B7H3, TIM-3, CD28, CD137, and OX40L were upregulated in M-subtype tumors and showed positive correlations with EMT scores in both cohorts, implying a complex immunosuppressive TME of M-subtype OSCC (**Figures 6A, B**). Notably, compared to the E-subtype tumors, OX40L was the most significantly upregulated immune checkpoint (TCGA: logFC = -2.174; *p* = 3.33E-32; GSE41613: logFC = -1.984; *p* = 1.26E-08) in M-subtype tumors and had a highest correlation with EMT score (TCGA: r = -0.644; *p* = 2.57E-38; GSE41613: r = -0.667; *p* = 8.97E-14), indicating that OX40L might be a promising immunotherapeutic targets for M-subtype patients.

### The Distinct Prognostic Prediction Values of Immune Checkpoints in Clinical Outcomes of Each EMT-Subtype Patients

Next, the relationships between EMT subtypes and clinical characteristics of OSCC patients were explored. The clinical characteristics of patients’ age, gender, pathologic T stage, pathologic N stage, pathologic tumor stage (TNM stage), smoking, alcohol, death, and progression were included. As **Supplementary Table 4** shows, EMT-subtype was linked to the pathologic T stage in the TCGA cohort and death in the GSE41613 cohort. However, no common clinical parameter showed any correlation with EMT-subtypes in both cohorts.

Then, we explored the prognostic implications of all immune checkpoints in OSCC patients in the TCGA cohort. Receiver operating characteristic (ROC) curve analysis was used to select the best cut-off value for each gene. The points of the maximum sum of sensitivity and specificity were selected. The results implied that high levels of PD-L1 and VISTA exhibited poor overall survival in OSCC patients, while high levels of CTLA4, BTLA, OX40, ICOS, and CD28 showed favorable survival. Remarkably, a perceptible difference of the prognostic prediction values was seen in each EMT subtype. The prognostic prediction values of PD-L1, CTLA4, and BTLA4 in all OSCC patients were not seen in E-subtype patients, whereas CD137 and CD137L showed significant correlations with E-subtype patients’ OS. High levels of HVEM, which showed no obvious correlation with OS in either all OSCC patients or E-subtype patients, predicted a poor OS in M-subtype ones (**Supplementary Figures 3, 4**). Collectively, these data implied that the heterogeneous immunity between E- and M-subtypes could influence patients’ clinical outcomes.

### A Novel Prognostic Model Named EIT Was Developed in OSCC

The TNM staging system remains the key determinant for prognostic prediction and risk stratification for treatment decisions in OSCC, despite its insufficiency in identifying tumor heterogeneity (2, 33). Given that the expression levels of immune checkpoints were correlated with EMT scores, and demonstrated potential impacts on OSCC patients’ survival, we questioned whether combining these parameters with TNM stages would yield further prognostic insight. Hence, we constructed a prognostic model named EIT based on EMT score, seven immune checkpoints mRNA levels (CTLA4, PD-L1, PD-L2, CD28, TIM-3, OX40L, and VISTA), and TNM stages. We randomly classified OSCC patients from TCGA datasets into two groups: training (n = 147) and validation (n = 143) cohorts. The total TCGA and GSE41613 cohorts were also used to validate the prognostic values of the EIT model. The ROC curves analysis was applied to generate an optimal cut-off value to separate patients into low-risk and high-risk groups in all cohorts.

**Figure 7:**
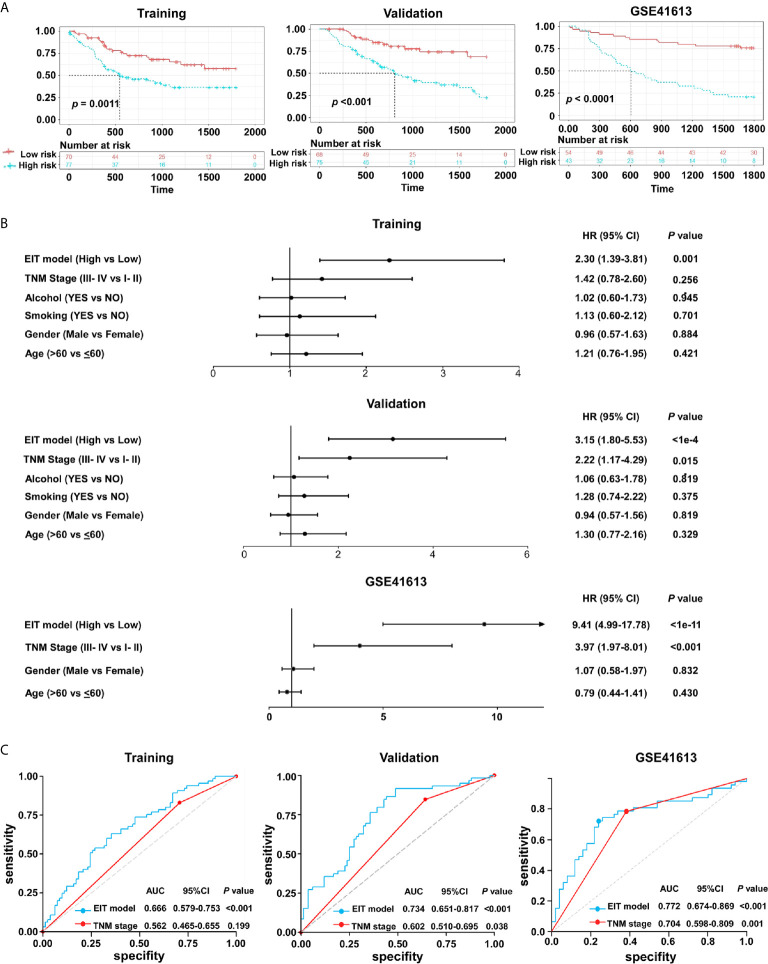
The EIT classifier is a novel prognostic prediction model in OSCC. **(A)** The Kaplan-Meier plots of overall survival according to the EIT model. *P* value was calculated by log-rank test. **(B)** Univariate association of the EIT model and clinicopathological characteristics with overall survival. **(C)** The ROC curve and AUC for the EIT model and TNM stage.

In the training cohort, 77/147 (52.4%) patients were assigned to the high-risk group and 70/147 (47.6%) patients were assigned to the low-risk group. In the validation cohort, 75/143 (52.4%) patients were assigned to the high-risk group and 68/143 (47.6%) patients were assigned to the low-risk group. We found that patients with high-risk had poorer 5-year OS than those with low-risk in both training and validation cohorts (**Figure 7A**). To further validate our findings, we also used the EIT model in the total TCGA and GSE41613 cohorts. We confirmed that in contrast to patients in the low-risk group, patients in the high-risk group exhibited shorter OS in both cohorts (**Figure 7A** and **Supplementary Figure 5A**).

The univariate analysis validated that the EIT model and TNM stages were substantially associated with OSCC patients’ overall survival (**Figure 7B** and **Supplementary Figure 5B**). After multivariable adjustment by clinicopathological variables, the EIT remained a strong independent prognostic factor for overall survival (**Table 1**). Then, we calculated the area under the receiver-operator characteristic (ROC) curve (AUC) to determine the sensitivity and specificity of the EIT model for predicting patients’ death. Meaningfully, the results confirmed that the combination of EMT score, immune checkpoints expression levels, and clinical characteristics improved our ability to predict OSCC patients’ clinical outcomes (training cohort: AUC, 0.666; 95% confidence intervals (CI), 0.579-0.753; validation cohort: AUC, 0.734; 95% confidence intervals (CI), 0.651-0.817; total TCGA cohort: AUC, 0.658; 95% confidence intervals (CI), 0.596-0.721; GSE41613 cohort: AUC, 0.772; 95% CI, 0.674-0.869) (**Figure 7C** and **Supplementary Figure 5C**). Collectively, our novel prognostic EIT model demonstrated that the TNM staging system combined with the biological features of EMT subtypes had delightful efficiency in predicting OSCC patients’ clinical outcomes.

**Table 1:** Multivariate Cox regression analysis of OSCC patients in the TCGA and GSE41613 datasets.

Variable	Training			Validation			GSE41613		
	HR	95% CI	*P* value	HR	95% CI	P value	HR	95% CI	*P* value
Age	1.331	0.810-2.187	0.258	1.089	0.615-1.927	0.770	1.432	0.758-2.706	0.268
Gender	1.146	0.641-2.052	0.646	0.998	0.558-1.786	0.995	1.021	0.542-1.923	0.949
Alcohol	1.356	0.766-2.401	0.297	0.891	0.503-1.579	0.692			
Smoking	1.008	0.523-1.940	0.982	1.356	0.759-2.423	0.303	/	/	/
EIT model	2.368	1.402-3.998	**0.001**	3.079	1.720-5.510	**<0.001**	10.757	5.419-21.351	**<0.001**

We calculated hazard ratios and P values with an adjusted multivariate Cox proportional hazards regression model, including age (>60 vs. ≤60), gender (male vs. female), alcohol (yes vs. no), smoking (former & current smoker vs. non-smoker), TNM stages (III-IV vs. I-II), and EIT model (high risk vs. low risk). The bold values mean that the difference is significant.

## Discussion

Accumulating evidence has demonstrated that cancer patients with an endogenous immune response that coexists with immune checkpoint elevation might be sensitive to the immune checkpoint agents. Given the increasing data shedding light on the correlation between EMT and tumor immune response, in the current study, a molecules classifier according to the EMT signature was used to divide OSCC patients into E- and M-subtypes. Tumors with E- and M-subtypes exhibited distinct immune microenvironments. M-subtype tumors were characterized as having an immunosuppressive TME with less active and more inactive or suppressive immune cells infiltration, as well as high expressions of immune checkpoints than E-subtype ones. Prominently, we developed and validated a novel prognostic classifier-EIT pattern which could improve the predicting efficiency of the current TNM staging system in OSCC.

Considering the wide range of cellular features that are influenced by the shifts along the epithelial-mesenchymal axis, it is not surprising that it influences the response to cancer immunotherapy strategies (34, 35). Malignant cells could induce EMT and release immunosuppressive signaling to create an immunosuppressive microenvironment *via* crosstalk with TME. In turn, immune cells could drive the EMT process in cancer cells through secreting cytokines and chemokines (13, 16). Thus, growing studies focused on the relationships between EMT status and immune landscapes (22, 25, 36). To explore the relationship between EMT status and immune landscapes in OSCC, we firstly tested the performance of an EMT signature in OSCC patients in the TCGA cohort and validated it in the GSE41613 cohort, which perfectly classified patients into E- and M-subtypes. The ESTIMATE analysis identified that the main source of EMT signature genes in OSCC bulk tissues was the TME. Remarkably, our findings are similar with the finding of scRNA-seq in OSCC, which demonstrated that M-subtype OSCC tumors mainly reflect TME components (30).

Currently, the general existence of distinct immune landscapes between epithelial and mesenchymal tumors have been noticed in lung cancer and urothelial cancer (22, 36). For instance, in lung cancer, the “mesenchymal” phenotype is associated with distinct TME changes, including the elevation of immune checkpoint molecules and the enhanced tumor infiltration by CD4+Foxp3+ regulatory T cells and CD3+ T cells (36). So far, the definite alteration of immune responses between E- and M-subtype OSCC is unknown. Here, we employed multiple methods, including KEGG, GO, ssGSEA, ESTIMATE, and CIBERSORT, to comprehensively illustrate the differences of cell components enrichment and molecular functions in E-subtype and M-subtype OSCC. In contrast to tumors with the E-subtype, the amount of immune cell subsets and the expressions of immune response signatures were all highly enriched in M-subtype OSCC, such as the signatures of 13 T cells which were correlated with CD8+ T cell infiltration (37), immune signaling molecule signatures which were correlated with the activation of the immune signaling pathway (38), and TLS signatures which were developed at sites of chronic inflammation and correlated with poor prognosis (39). Therefore, we implied that M-subtype OSCC was defined as “immune hot” tumors.

The tumor-immunity cycle forms the intellectual framework for immune-based therapeutic strategies (40, 41). Broadly, antigen presenting cells (APCs) like dendritic cells (DCs), macrophages, and mast cells uptake the tumor antigens to bind to MHC molecules, then, travel to the lymphoid organs to generate CD8+ cytotoxic T effector cells (CTLs). CTLs migrate to the tumor site, product cytotoxic effectors, and kill tumor cells. Besides, B lymphocytes differentiate into plasma cells and produce antitumor antibodies. DCs predominantly activate CD4+ T helper cells, which ensure the amplification of T cell response without deleterious autoimmunity. Generation of memory immune cells is important for long-term immune response (42). Therefore, the crosstalk between different immune cell types need to be precisely regulated, on which damage can lead to immunodeficiency and promote cancer progression. As tumor-associated macrophages have been reported to be the major constituent of the TME and are correlated with more aggressive subtypes, stronger chemotherapy resistance, and worse clinical outcome in many cancers (43), we also found that macrophages and T cells were confirmed to be the prominent infiltrated immune cells in OSCC specimens. Furthermore, more inactivation or suppression of immune cells (M0 and M2 macrophages and resting memory CD4+ T cells), and less active immune cells (CD8+ T cells, activated memory CD4+ T cells, TFH cells, NK cells, activated DCs, and plasma cells) were found in M-subtype OSCC tumors than E-subtype ones in TCGA or GSE41613 datasets, indicating an immunosuppression TME in M-subtype patients.

Currently, cancer immunotherapy is mainly focused on leveraging the cytotoxic potential of tumor-​specific CTLs (44, 45). Among all the immune-based anti-cancer strategies, immune checkpoint blockade has had the broadest impact, which has been applied to many cancers with promising results, such as advanced melanoma (46), non-small-cell lung cancer (47), and HNSC (6, 7). Nevertheless, few relationships between patients’ overall survival and PD-L1 expression levels have been identified in HNSC patients who were treated with anti-PD1 antibodies, highlighting that a broader measure of the tumor microenvironment is needed to understand the intrinsic resistance mechanisms, identify prediction biomarkers, and select ideal subgroups for this kind of therapy (6, 10). In this study, we confirmed that most of the altered immune checkpoints were highly expressed in M-subtype OSCC in TCGA datasets. Yet, in the GSE41613 dataset, only B7H3, TIM3, OX40L, CD28, and CD137 were validated to be significant, implying a striking heterogeneity in different patient populations. PD-1 and PD-L1 showed no significant difference in each OSCC EMT phenotype. Since the co-expression of multiple immune checkpoints has been commonly seen on exhausted T cells in the TME, M-subtype OSCC patients with multiple elevations of immune checkpoints were further verified to have an immunosuppressive feature. Prominently, growing evidence indicated that patients with inflamed tumors would likely benefit from anti-PD-1 therapy. Furthermore, the IFN-γ signature genes which were highly expressed in M-subtype OSCC were found to be significantly associated with survival with R/M-HNSCC in the KEYNOTE-040 trial. Thus, these data demonstrated that M-subtype OSCC patients who have both an endogenous immune response and immune checkpoint elevation might be more sensitive to the immune checkpoint agents in contrast to E-subtype patients.

Appropriate treatment choices are based on accurate prognostic assessments. Current classification of OSCC based on the TNM staging system fails to capture biologic heterogeneity or adequately inform treatment (2, 33). Given that EMT signatures have a profound effect on the TME in human cancer, it is not surprising to find that the combination of EMT-related gene expression and tumor-infiltrating T cell abundance have a disparate impact on survival in urothelial cancer patients (22). Our findings also demonstrated a striking association between EMT signature and immune checkpoints expressions. Thus, we developed a prognostic model named EIT based on EMT signature, immune checkpoints expressions, and TNM stage. We demonstrated that patients with high EIT score exhibited poor clinical outcomes in all cohorts. More critically, our novel prognostic classifier significantly improved the efficiency of TNM stage in predicting the overall survival of OSCC patients.

## Data Availability Statement

The original contributions presented in the study are included in the article/**Supplementary Material**. Further inquiries can be directed to the corresponding authors.

## Author Contributions

TW, BC, S-YZ, X-YR, and C-YW designed the research. S-YZ, X-YR, C-YW, X-JC, R-YC, QL, XP, J-YZ, W-LZ, and X-RT conducted the bioinformatic analysis and acquired the data. TW, BC, S-YZ, X-YR, and C-YW wrote the manuscript. All authors contributed to the article and approved the submitted version.

## Funding

This work was supported by grants from the National Natural Science Foundation of China (81702700, 81700979, 81903134); the Postdoctoral Science Foundation of China (2019M653231); the Guangdong Financial Fund for High-Caliber Hospital Construction (174-2018-XMZC-0001-03-0125/C-08); the Natural Science Foundation of Guangdong Province (201A1515010679, 2019A1515011427); the Science and Technology Program of Guangzhou city of China (201804010144); and the Fundamental Research Funds for the Central Universities (19ykpy83). The funders had no role in the study design, data collection, analysis, decision to publish, or the preparation of the manuscript.

## Conflict of Interest

The authors declare that the research was conducted in the absence of any commercial or financial relationships that could be construed as a potential conflict of interest.
