# Supplementary material for: Comprehensive Characterization of Immune Landscape Based on Epithelial-Mesenchymal Transition Signature in OSCC: Implication for Prognosis and Immunotherapy
Source: Front Oncol. 2021 Jul 1;11:587862. doi: 10.3389/fonc.2021.587862 (PMC8281347; doi:10.3389/fonc.2021.587862)
Supplement: Supplementary file 1 [file DataSheet_1.docx]

**Title:**

**Comprehensive characterization of immune landscape based on epithelial-mesenchymal-transition signature in OSCC: implication for prognosis and immunotherapy**

**Authors and Affiliations:**

Si-yuan Zhang,^1*^ Xian-yue Ren,^1*^ Chun-yang Wang,^1*^ Xi-juan Chen,^1^ Ruo-yan Cao,^1^ Qin Liu,^1^ Xue Pan,^1^ Jia-ying Zhou,^1^ Wei-lin Zhang,^1^ Xin-Ran Tang,^2^ Bin Cheng^1#^ and Tong Wu^1#^

^1^ Guangdong Provincial Key Laboratory of Stomatology, Guanghua School of Stomatology, Hospital of Stomatology, Sun Yat-sen University, Guangzhou, Guangdong, 510055, China.

^2^ Department of Radiation Oncology, Nanfang Hospital, Southern Medical University, Guangzhou, Guangdong, 510515, China.

**^*^ Si-yuan Zhang, Xian-yue Ren and Chun-yang Wang contributed equally to this article.**

**^#^Corresponding authors:**

**Tong Wu,** Guangdong Provincial Key Laboratory of Stomatology, Guanghua School of Stomatology, Hospital of Stomatology, Sun Yat-sen University, Guangzhou 510055, Guangdong, People’s Republic of China; **Telephone:** 020-83862558; **Fax:** 020-83822807; **E-mail:** wutong23@mail.sysu.edu.cn

**Bin Cheng**, Guangdong Provincial Key Laboratory of Stomatology, Guanghua School of Stomatology, Hospital of Stomatology, Sun Yat-sen University, Guangzhou 510055, Guangdong, People’s Republic of China; **Telephone:** 020-83862558; **Fax:** 020-83822807; **E-mail:** chengbin@mail.sysu.edu.cn


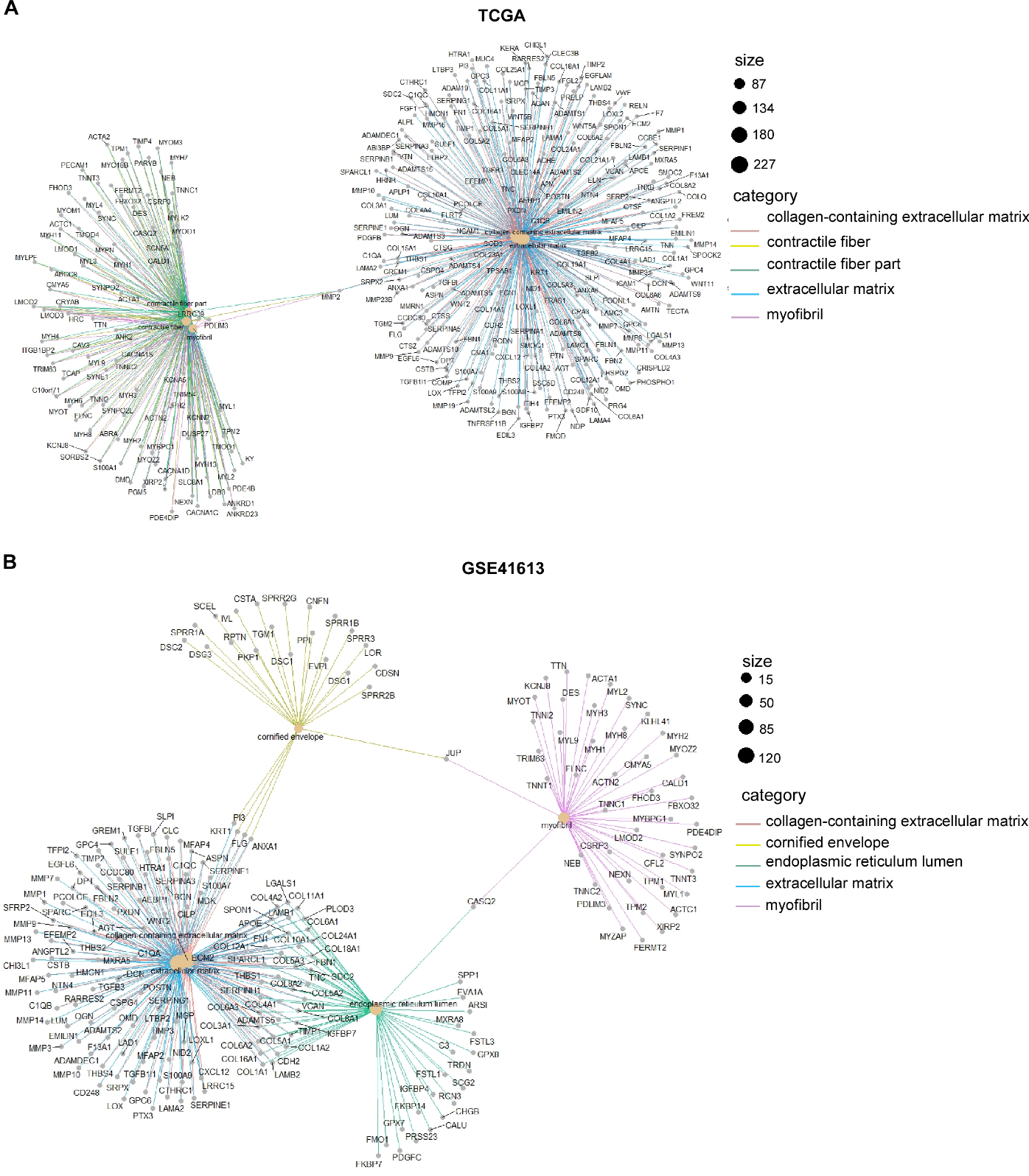


**Supplementary Figure 1.** The enrichment of differentially expressed genes between E- and M- subtypes is determined by the cell component of GO analysis in **(A)** TCGA and **(B)** GSE41613 dataset.


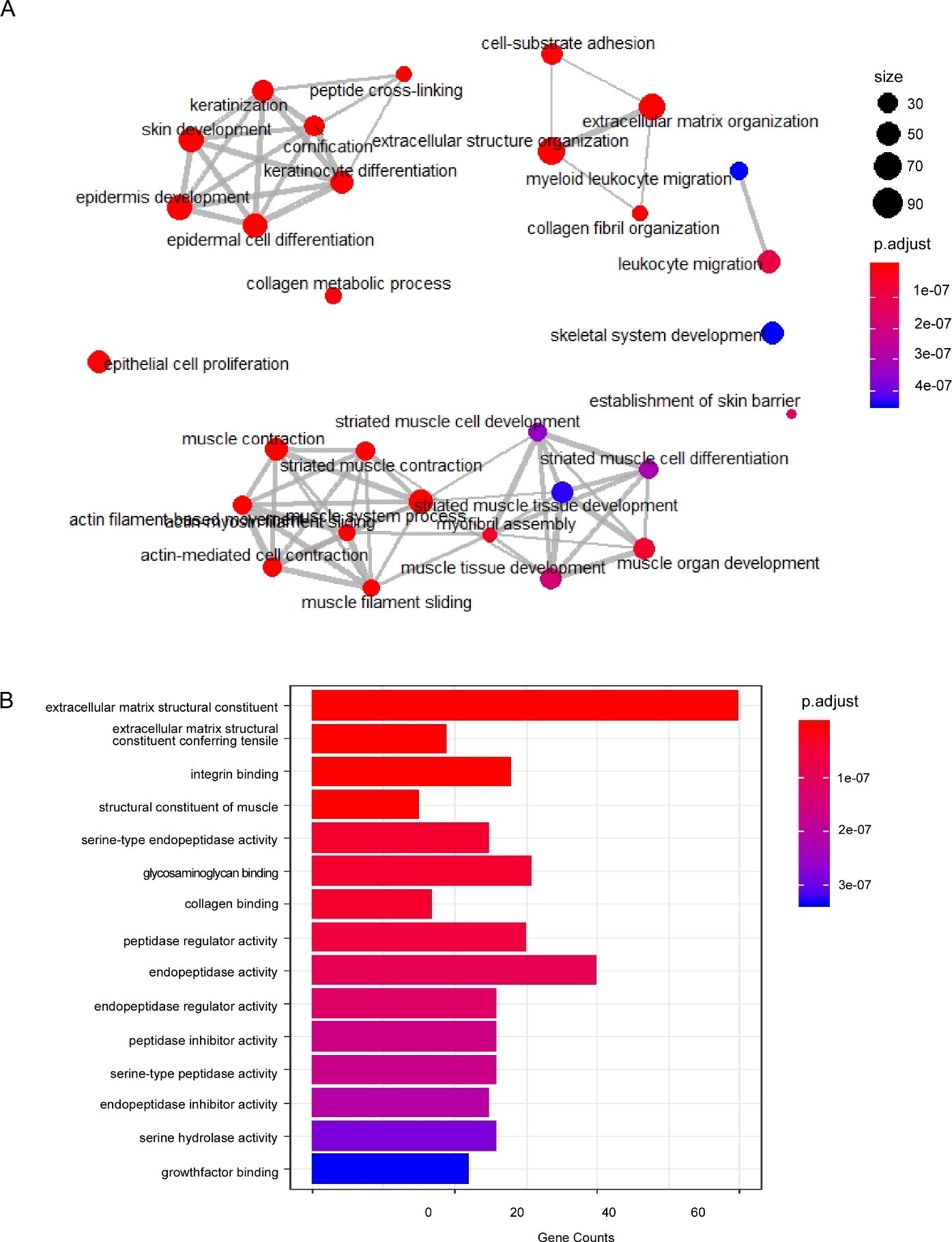


**Supplementary Figure 2.** The enrichment of differentially expressed genes between E- and M- subtypes is determined by the **(A)** biological process and **(B)** molecular function cell component of GO analysis in GSE41613 dataset.


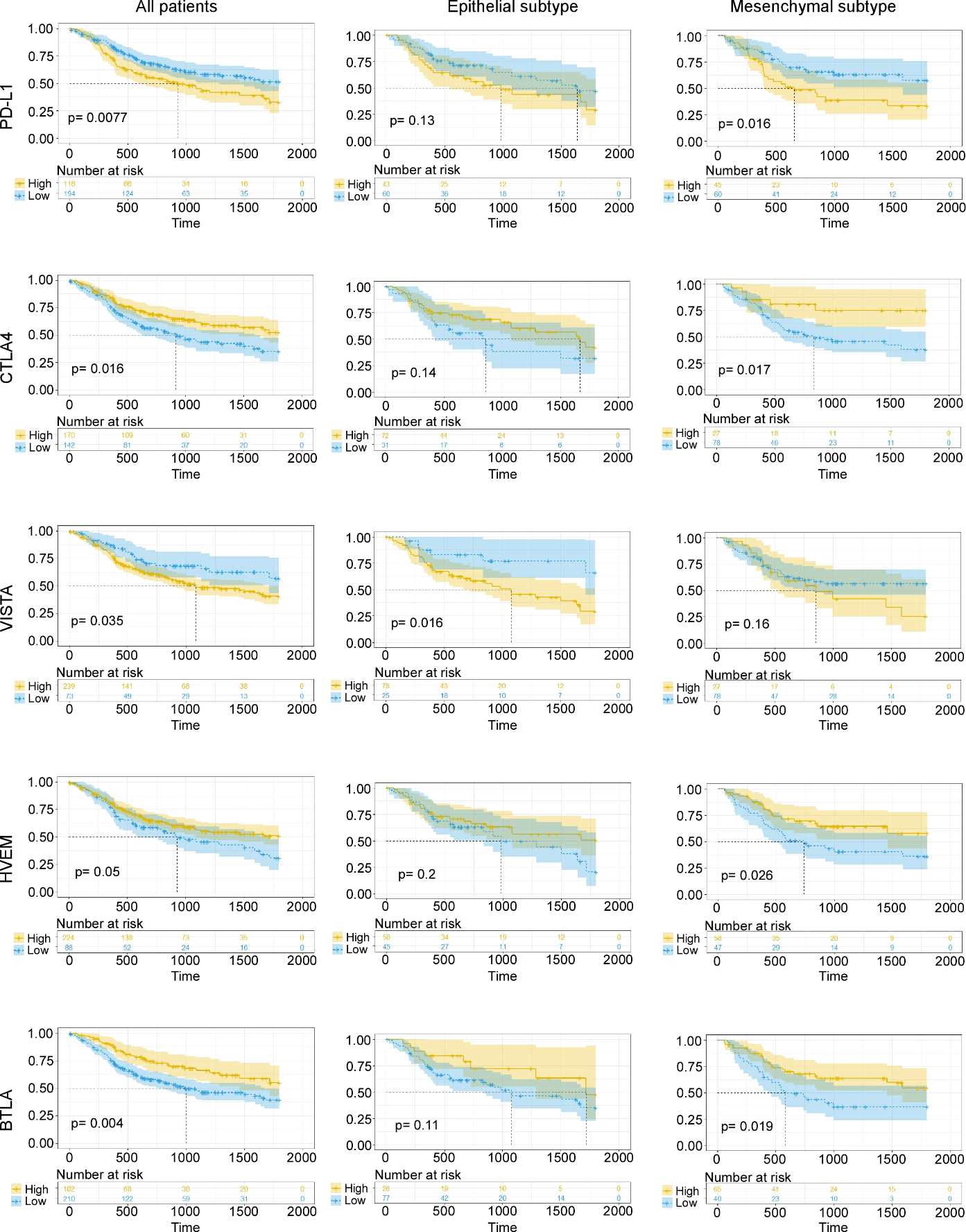


**Supplementary Figure 3.** Kaplan-Meier plots of overall survival according to the co-inhibitory immune checkpoints’ expression levels in all patients (n = 312), E-type patients (n = 103) and M-type (n = 105) patients using TCGA dataset.


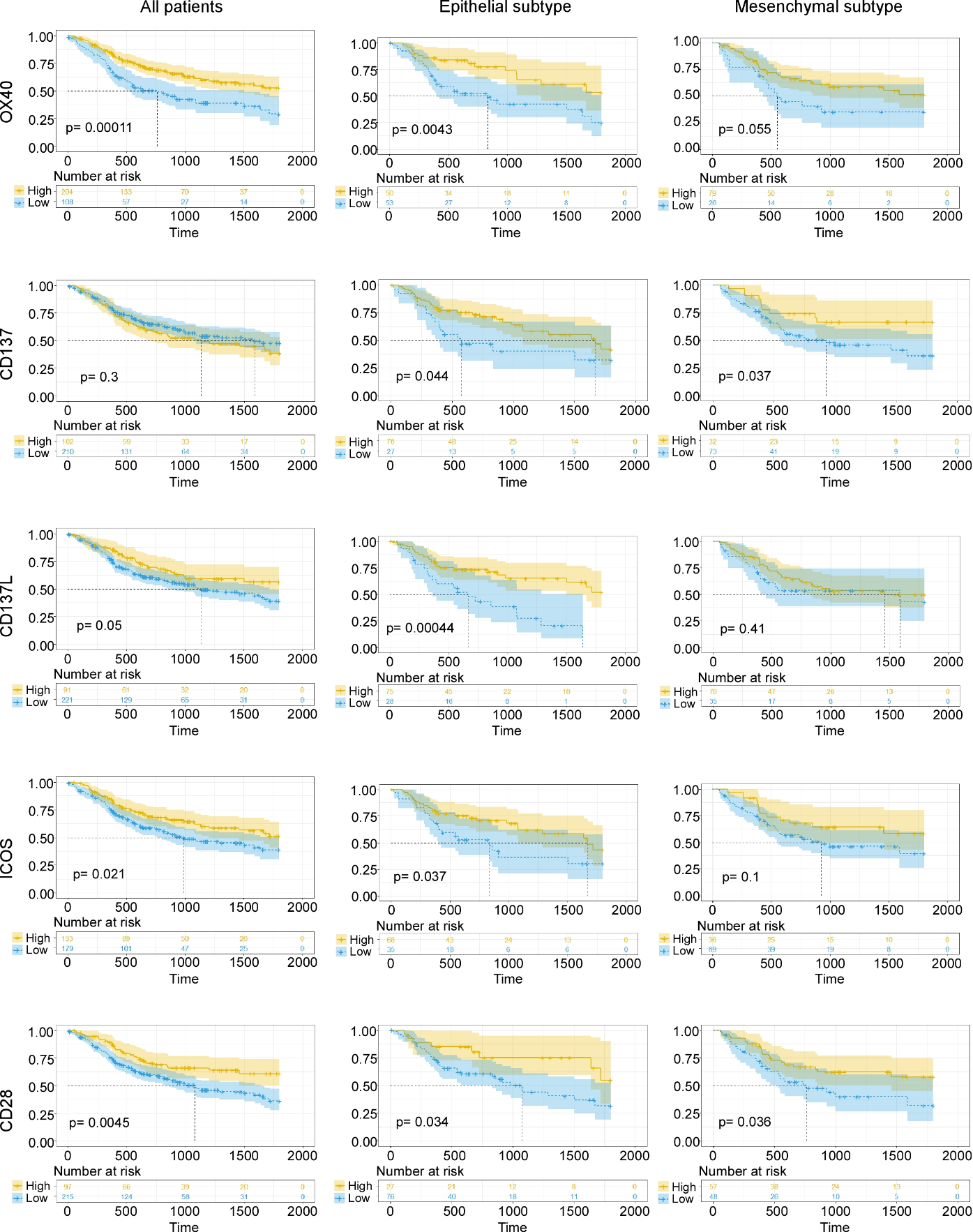


**Supplementary Figure 4.** Kaplan-Meier plots of overall survival according to the co-stimulatory immune checkpoints’ expression levels in all patients (n = 312) , E-type patients (n = 103) and M-type (n = 105) patients using TCGA dataset.


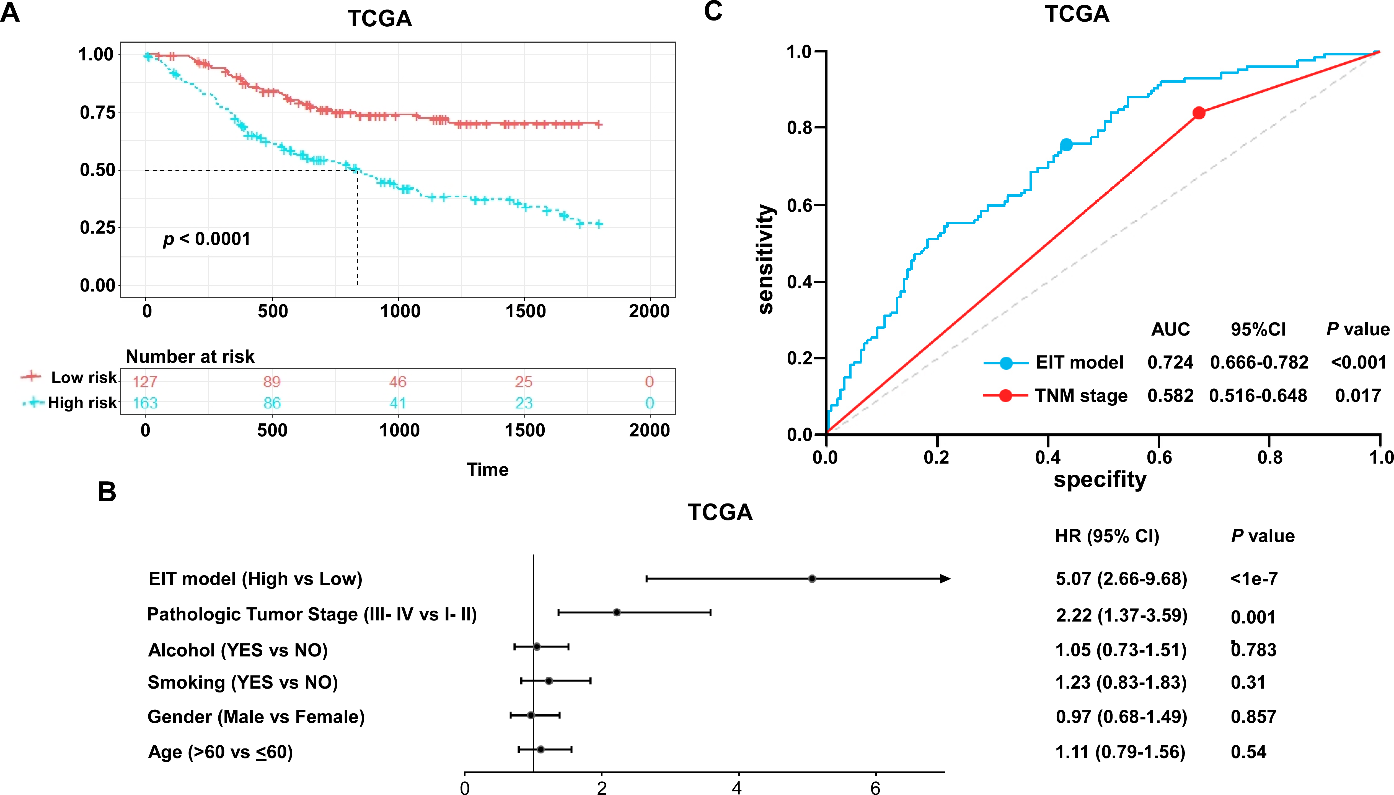


**Supplementary Figure 5. The EIT classifier is a novel prognostic prediction model in OSCC.** Computer generated random numbers were used to distribute patients from TCGA into training and validation cohorts. **(A)** The Kaplan-Meier plots of overall survival according to EIT model in training cohort (n = 147) and validation cohort (n = 143). *P* value was calculated by log-rank test. **(B)** Univariate association of EIT model and Clinicopathological characteristics with overall survival in training cohort and validation cohort. **(C)** The ROC curve and AUC for the EIT model and TNM stage in training cohort and validation cohort.

**Supplementary Table 2. Publicly available gene signatures used in the study.**

| **Signature Name** | **Reference** |
| --- | --- |
| Immune enrichment score | Yoshihara et al. Nat Commun. 2013;4:2612 |
| Stromal enrichment score | Yoshihara et al. Nat Commun. 2013;4:2612 |
| Immune cell subsets | Cancer Genome Atlas Network. Cell. 2015;161:1681-96 |
| Immune signaling molecules | Cancer Genome Atlas Network. Cell. 2015;161:1681-96 |
| 13 T-cell signature | Spranger et al. Proc Natl Acad Sci U S A. 2016;113(48):E7759-E7768. |
| Tertiary Lymphoid Structure | Finkin et al. Nat Immunol. 2015;16:1235-44 |
| 6-gene IFN signature | Chow et al. J Clin Oncol. 34, (suppl; abstr 6010) 2016 |

**Supplementary Table 3. Estimation of the proportions of leukocyte compositions using CIBERSORT in the TCGA and GSE41613 database.**

|  | TCGA | | |  | GSE41613 | |  |
| --- | --- | --- | --- | --- | --- | --- | --- |
| Cell types | E- type (%) | M-type (%) | P-value^*^ | | E- type (%) | M-type (%) | P*-*value^*^ |
|  | n = 105 | n = 105 |  |  | n = 32 | n = 32 |  |
| **Macrophages** | 42.874 | 51.783 | **<0.001** | | 32.134 | 39.74 | **0.010** |
| M0 | 23.397 | 29.962 | **0.003** | | 14.064 | 23.700 | **<0.001** |
| M1 | 9.135 | 8.079 | 0.301 | | 11.112 | 9.275 | 0.269 |
| M2 | 10.342 | 13.742 | **<0.001** | | 6.958 | 6.765 | 0.839 |
| **T cells** | 12.027 | 8.212 | 0.162 | | 29.199 | 28.711 | 0.871 |
| CD8 | 5.432 | 3.154 | **0.010** | | 3.604 | 2.977 | 0.586 |
| CD4.naive | 0.421 | 0.043 | 0.073 | | 3.080 | 3.227 | 0.887 |
| CD4.memory.resting | 9.014 | 13.480 | **<0.001** | | 5.120 | 6.978 | **0.027** |
| CD4.memory.activated | 2.195 | 1.385 | **0.037** | | 7.624 | 6.484 | 0.408 |
| follicular.helper | 8.595 | 6.011 | **<0.001** | | 4.188 | 2.801 | **0.044** |
| regulatory..Tregs. | 3.966 | 3.623 | 0.454 | | 0.139 | 0.013 | 0.209 |
| gamma.delta | 0.013 | 0.007 | 0.666 | | 5.444 | 6.231 | 0.597 |
| **NK cells** | 7.534 | 6.533 | **0.027** | | 1.937 | 1.215 | 0.083 |
| resting | 6.657 | 5.719 | 0.053 | | 1.524 | 1.109 | 0.324 |
| activated | 0.877 | 0.814 | 0.796 | | 0.413 | 0.106 | 0.101 |
| **Dendritic cells** | 6.155 | 4.208 | **0.014** | | 6.94 | 3.622 | **0.005** |
| resting | 1.437 | 1.055 | 0.294 | | 3.850 | 2.053 | **0.010** |
| activated | 4.718 | 3.153 | **0.018** | | 3.090 | 1.569 | 0.137 |
| **Mast cells** | 5.661 | 4.499 | **0.045** | | 10.264 | 12.058 | 0.387 |
| resting | 2.895 | 2.406 | 0.263 | | 2.104 | 1.487 | 0.476 |
| activated | 2.766 | 2.093 | 0.278 | | 8.160 | 10.571 | 0.301 |
| **B cells** | 1.117 | 1.486 | 0.334 | | 1.966 | 1.615 | 0.571 |
| naive | 0.989 | 1.302 | 0.407 | | 0.271 | 1.088 | 0.061 |
| memory | 0.128 | 0.184 | 0.459 | | 1.695 | 0.527 | **0.012** |
| **Plasma.cells** | 5.574 | 2.311 | **<0.001** | | 12.461 | 7.929 | **0.039** |
| **Monocytes** | 0.787 | 0.719 | 0.768 | | 0.101 | 0.016 | 0.243 |
| **Eosinophils** | 0.098 | 0.093 | 0.907 | | 0.427 | 0.896 | 0.163 |
| **Neutrophils** | 0.559 | 0.664 | 0.680 | | 4.570 | 4.196 | 0.715 |

*Chi-square test or Fisher’s exact test.

**Supplementary Table 4. Correlations between EMT subtypes and clinical features in patients with OSCC from the TCGA and GSE41613 datasets.**

|  | TCGA | | P-value^*^ | GSE41613 | | P*-*value^*^ |
| --- | --- | --- | --- | --- | --- | --- |
| Characteristic | E- type (%) | M-type (%) |  | E- type (%) | M-type (%) |  |
|  | n = 105 | n = 105 |  | n = 32 | n = 32 |  |
| Age (median) |  |  |  |  |  |  |
| ≤60 | 49 (46.7) | 52 (49.5) | 0.782 | 16（50.0） | 16（50.0） | 1 |
| >60 | 56 (53.3) | 53(50.5) |  | 16（50.0） | 16（50.0） |  |
| Gender |  |  |  |  |  |  |
| Male | 29 (27.9) | 36 (34.3) | 0.371 | 10（31.3） | 13（40.6） | 0.603 |
| Female | 75 (72.1) | 69 (65.7) |  | 22（68.8） | 19（59.4） |  |
| Pathologic T |  |  |  |  |  |  |
| T1-2 | 29 (29.6) | 47 (47.0) | **0.013** |  |  |  |
| T3-4 | 69 (70.4) | 53 (53.0) |  |  |  |  |
| Pathologic N |  |  |  |  |  |  |
| N0-1 | 89 (97.8) | 88 (100.0) | 0.497 |  |  |  |
| N2-3 | 2 (2.2) | 0 (0) |  |  |  |  |
| Pathologic Tumor Stage |  |  |  |  |  |  |
| Stage I- II | 4 (9.3) | 8 (22.3) | 0.129 | 15（46.9） | 11（34.4） | 0.446 |
| Stage III- IV | 39 (90.7) | 28 (77.7) |  | 17（53.1） | 21（65.6） |  |
| Smoking |  |  |  |  |  |  |
| Non-smoker | 33 (32.4) | 31 (30.4) | 0.763 |  |  |  |
| Former & current smoker | 69 (67.6) | 71 (69.6) |  |  |  |  |
| Alcohol |  |  |  |  |  |  |
| No | 41 (40.2) | 30 (29.1) |  |  |  |  |
| Yes | 61 (59.8) | 73 (70.9) |  |  |  |  |
| Death |  |  |  |  |  |  |
| No | 56 (53.3) | 56 (53.3) | 0.938 | 22（68.8） | 13（40.6） | **0.047** |
| Yes | 49 (46.7) | 49 (46.7) |  | 10（31.3） | 19（59.4） |  |
| Progression |  |  |  |  |  |  |
| No | 71 (67.6) | 73 (69.5) | 0.882 |  |  |  |
| Yes | 34 (32.4) | 32 (30.5) |  |  |  |  |

*Chi-square test or Fisher’s exact test.
